# Experimental Evaluation of the Packet Reception Performance of LoRa

**DOI:** 10.3390/s21041071

**Published:** 2021-02-04

**Authors:** Qingjie Guo, Fengxu Yang, Jianming Wei

**Affiliations:** 1 Shanghai Advanced Research Institute, Chinese Academy of Sciences, Shanghai 201210, China; guoqj@shanghaitech.edu.cn (Q.G.); yangfx@shanghaitech.edu.cn (F.Y.); 2 University of Chinese Academy of Sciences, Beijing 100049, China; 3 School of Information Science and Technology, ShanghaiTech University, Shanghai 201210, China

**Keywords:** IoT technology, LoRa, physical layer parameters, packet length, transmission scheme

## Abstract

LoRa technology is currently one of the most popular Internet of Things (IoT) technologies. A substantial number of LoRa devices have been applied in a wide variety of real-world scenarios, and developers can adjust the packet reception performance of LoRa through physical layer parameter configuration to meet the requirements. However, since the important details of the relationship between the physical layer parameters and the packet reception performance of LoRa remain unknown, it is a challenge to choose the appropriate parameter configuration to meet the requirements of the scenarios. Moreover, with the increase in application scenarios, the requirements for energy consumption become increasingly high. Therefore, it is also a challenge to know how to configure the parameters to maximize the energy efficiency while maintaining a high data rate. In this work, a complex evaluation experiment on the communication capability under a negative Signal to Noise Ratio is presented, and the specific details of the relationship between physical layer parameters and the packet reception performance of LoRa are clarified. Furthermore, we study the impact of the packet length on the packet reception performance of LoRa, and the experimental results show that when there is a large amount of data to be transmitted, it is better to choose long packets instead of short packets. Finally, considering the influence of physical layer parameters and the packet length on the packet reception performance of LoRa, the optimal parameter combination is explored, so as to propose a transmission scheme with a balanced reliability, delay, and energy consumption. This scheme is the first to consider the physical layer parameters and packet length together to study the communication transmission scheme, which reduces the communication time by 50% compared with the traditional transmission scheme and greatly reduces the energy consumption.

## 1. Introduction

In order to make our way of life more efficient, the industrialized world is becoming increasingly automated. Various innovative technologies are constantly being proposed. The Internet of Things (IoT) is currently one of the most popular innovative technologies. The technologies related to the IoT are continuing to develop, and the number of IoT devices is also rapidly increasing. The Ericsson Mobility Report pointed out that IoT connected devices will grow from 7 billion in 2017 to 20 billion in 2023 [1]. At present, the practical application of the IoT has achieved remarkable results in many fields, such as manufacturing, agriculture [2], home furnishing [3], transportation, car networking, and medical treatment [4]. However, in order to connect IoT devices, wireless networks are required to provide reliable communication and a higher energy efficiency coverage [5]. The above requirements have led to a simpler technical solution called the “Low Power Wide Area Network (LPWAN)”, which can provide low-energy communication over a distance of several kilometers [6,7]. Therefore, for long-distance applications such as sensor networks, LPWAN technology occupies an absolute dominant position.

LoRa [8,9] is a kind of LPWAN communication technology and is a long-distance wireless transmission solution based on spread spectrum transmission techniques and chirp spread spectrum (CSS) modulation. LoRa’s low power consumption, long transmission distance, flexible networking capability, and many other features fit perfectly with the needs of IoT fragmentation, providing a low cost and many connections [10,11,12]. Therefore, LoRa is widely deployed in multiple scenarios, such as campuses [13,14], buildings [15], and forest farms [16], and has broad prospects for future development [17]. Meanwhile, this also results in higher requirements for the performance of LoRa (such as reliability, delay, and energy consumption). In order to improve the communication capability between LoRa nodes and meet the requirements of a wide variety of scenarios, developers need to know the important details of the relationship between physical layer parameters and the packet reception performance of LoRa.

LoRa is a chirp-modulated high-order M-ary FSK system. The modulation and demodulation process is described by rigorous mathematical signal processing in [18]. Comparing the results of FSK modulation and LoRa modulation in the additive white Gaussian noise channel shows that the performances of the two are similar. Furthermore, the results of Brecht and Sofie [19], who compared the performance of LoRa modulation and BPSK modulation in the additive white Gaussian noise channel, demonstrate that LoRa modulation has better interference immunity, because LoRa modulation occupies more bandwidth. However, the authors [18,19] did not consider the influence of channel coding and the model proposed by the authors [19] only considers the influence of Spreading Factor (SF) parameters. In [20], the authors consider more factors that affect the packet reception performance of LoRa, including the Bandwidth (BW) and Coding Rate (CR). Their results indicated that LoRa technology can provide very robust communication, even when there is a high noise level. However, the authors did not consider a wide enough Signal to Noise Ratio (SNR) range, and the experimental result of the relationship between physical layer parameters and the packet reception performance of LoRa was not sufficiently detailed. As a result, the important details of the relationship between physical layer parameters and the packet reception performance of LoRa are still unclear.

Furthermore, LoRa devices have been deployed in various scenarios, and different scenarios will have different requirements for the performance of LoRa. Some scenarios require a higher communication reliability for LoRa devices, such as smart water metering, and others require LoRa devices to use less energy to complete communication, such as smart forest farms. The reliability of communication between LoRa nodes can be improved by adjusting the physical layer parameters, which has been demonstrated in [21]. The authors indicated that in order to maintain reliable communication, SF and CR should be configured to the highest values (a higher value means a higher receiving sensitivity). The experiment of Yim et al. [16], which deployed LoRa nodes on a forest farm, also obtained similar results. Meanwhile, other previous work has also tried to use a more sensitive parameter configuration to improve the reliability of communication and experimental results have shown that the packet reception ratio was improved. However, using the most sensitive parameter configuration will minimize the data rate, which will bring about an increase in the communication time and energy consumption, as well as shortening of the working time of the node. Obviously, designing a transmission scheme that minimizes the energy consumption while maintaining a high data rate, thereby balancing reliability, delay, and energy consumption, is essential for successful deployment in real-world scenarios. 

In this study, we conducted extensive experiments to study the reliability of the communication link between LoRa nodes when the channel is interfered by white Gaussian noise, and we propose a transmission scheme that can achieve a balance between reliability, delay, and energy efficiency. To begin with, we studied the relationship between physical layer parameters (SF, BW, and CR) and the packet reception performance of LoRa in a sufficiently wide SNR range (from −24 to 0 dB) and obtained detailed experimental results. Our experimental results found that physical layer parameters have a great impact on the packet reception performance of LoRa, and based on this, the optimal parameter combination was discussed in relation to the packet reception performance of LoRa. Next, we conducted a theoretical analysis and extensive experiments to study the impact of the packet length on the packet reception performance of LoRa. Our experimental results show that the Packet Error Ratios of different packet lengths are nearly equal in areas where the Packet Error Ratio is less than 0.2, that is, different packet lengths hardly affect the packet reception performance of LoRa in these areas. However, in areas where the Packet Error Ratio is greater than 0.2, long packets reduce the packet reception performance of LoRa and drastically increase packet loss and corruption. It seems that a short packet transmission scheme would be a better choice. However, short packets mean more headers than long packets, and this cost should not be underestimated. Therefore, if a node needs to send a large amount of data, the transmission scheme should use long packets instead of short packets. Finally, in order to select the appropriate parameters to optimize the transmission scheme, we combined the previous experimental results with the retransmission mechanism to strike a balance between communication reliability, delay, and energy efficiency. The experimental results show that the time of successful communication between nodes is reduced by 50% compared with the traditional transmission scheme, and the energy consumption is also greatly reduced.

The contributions provided in this article can be summarized as follows:

(1) We studied the influence of physical layer parameters on the reliability of communication links between LoRa nodes, and obtained detailed experimental results on the relationship between the two;
(2) The influence of the packet length on the reliability of communication links between LoRa nodes was studied by a theoretical analysis and extensive experiments. It should be noted that it is not worth using short packets instead of long packets;
(3) We analyzed the influence of the physical layer parameter settings on the retransmission effect and proposed how to configure the physical layer parameters to minimize the energy consumption and maintain a high data rate, so as to achieve a balance between reliability, delay, and energy efficiency.

We discuss related work in Section 2. In Section 3, we introduce LoRa technology. We created a LoRa transceiver based on USRP B200 software radios and carried out LoRa performance evaluation experiments under white Gaussian noise interference. We used this transceiver as a transmitter of LoRa signals, and two commercial devices as receivers. The specific experimental environment and methods are introduced in Section 4. In Section 5, the results and analysis of the LoRa performance evaluation experiment under different parameters are presented. Firstly, we studied the influence of LoRa physical layer parameters on the LoRa performance. Secondly, we considered the impact of the packet length on the LoRa performance. Thirdly, we compared the performance of two commonly used commercial transceivers. Finally, for the scene of noise interference, we propose what parameters to choose to reduce the communication time and power consumption. We discuss future work in Section 6 and present the conclusions of this work in Section 7.

## 2. Related Work

In this section, we do our best to introduce related work on the impact of physical layer parameters on the performance of LoRa. In [22], the authors conducted extensive experimental research on the reliability of LoRa. At the same time, the authors also studied the influence of temperature on the performance of LoRa and found that high temperatures decrease the received signal strength and drastically increase packet loss and corruption for nodes at the edge of the communication range. However, the influence of several physical layer parameters (for example, SF8) on the performance of LoRa has not yet been determined. The paper [23] answered some common questions faced by system users and researchers regarding LoRa, and verified Semtech’s commitments in terms of the transmission distance, node life, and capacity. We also noticed that some experimental results on the performance of LoRa are not as good as Semtech claims. Yim et al. [16] measured the communication distance of LoRa on a forest farm and found that the actual communication range of LoRa was shorter than the theoretical communication range, and the reliability of the actual data was inconsistent with the report when employing different distances and physical layer parameter configurations. Additionally, the author also found that the Fresnel zone affects the LoRa network. The setting of physical layer parameters not only affects the performance of LoRa, but also the capacity of the LoRa network. The relationship between physical layer parameter settings and the capacity of the LoRa network is discussed in [24]. The researchers have shown, through simulation, that the choice of physical layer parameter settings affects the number of LoRa nodes that can access the channel at the same time, resulting in the limitation of the capacity of the LoRa network. Similar results were obtained in [14]. The work in [14] is also the first to propose the use of LoRa technology for health detection, and the authors have pointed out that if there are multiple devices in the same environment, there may be problems with the communication reliability. In [15], the author evaluates the communication performance of LoRa in a building by changing the transmit power, data rate, package length, and location of the wireless module. The experimental results show that the practical considerations of signal reliability in different building environments lead to certain limitations in practical applications. Therefore, it remains a challenge to improve the reliability while maintaining high data rates and low energy consumption. Meanwhile, LoRa technology uses different spreading factors to increase the number of nodes in the channel, which is the use of orthogonality of different spreading factors. However, the experimental results in [25] show that different spreading factors will also exhibit conflict and greatly reduce the performance of LoRa. Moreover, the author believes that the packet length is not a key factor affecting the performance of LoRa. Similarly, the results presented in this work show that there is no obvious difference in the Packet Error Ratio of using short packets or long packets, except at the edge of the communication link. Other works [21,26,27] have evaluated the performance of LoRa in different environments, and suggested using the most sensitive parameter configuration to improve the reliability of LoRa. However, they did not consider all parameter configurations, and important details on the relationship between physical layer parameters and the performance of LoRa are not sufficiently detailed and specific. In addition, although the transmission scheme they proposed has an improved reliability, the data rate and energy consumption have also increased accordingly.

Different from previous work, this article expects to obtain important details on the impact of physical layer parameters and packet length settings on the packet reception performance of LoRa. Furthermore, given the SNR, it will comprehensively consider the impact of physical layer parameters and the packet length on the performance of LoRa to optimize the parameter configuration, thereby reducing the time and energy consumption of successful communication and ultimately achieving a balance of reliability, delay, and energy consumption.

## 3. LoRa

**Figure 1:**
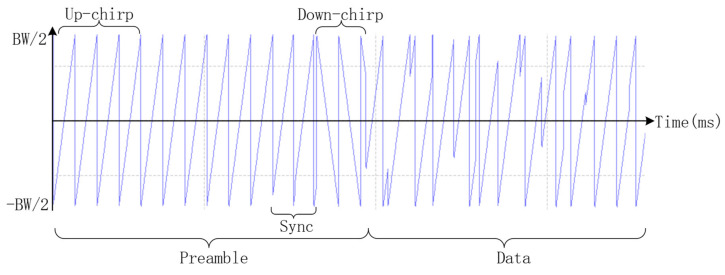
The LoRa packet structure.

LoRa is an ultra-long distance wireless transmission scheme based on spread spectrum technology from Semtech. LoRa mainly operates in license-free frequency bands around the world, including 433, 868, 915 MHz, etc. The LoRa structure includes preamble and data parts, as shown in Figure 1. The preamble part consists of several standard consecutive up-chirps, sync, and 2.5 down-chirps. Up-chirps always move in the same direction and with the same slope, until reaching the edge of the frequency band, while down-chirps exhibit the opposite slope. The data part is composed of chirps with different initial positions. We can see that different symbols lead to different initial positions, which indicates that the initial point of each chirp is used for data modulation. 

The Spreading Factor (SF), Bandwidth (BW), and Coding Rate (CR) are the modulation parameters of LoRa [28]. LoRa uses multiple chips to represent data from payload information. The transmission speed of spreading information is called the symbol rate (Rs), and the ratio of the chip rate to the nominal Rs is the SF, which indicates the number of symbols sent for each information bit. SF can range from 6 to 12. However, because SF6 requires a special configuration, it is generally not used. The number of chips per symbol is calculated as $2^{\mathrm{SF}}$. For example, with an SF of 7 (SF7), 128 chips/symbol are used. A lower SF means that more valid data can be sent per second, and a higher SF can only send a small amount of valid data. Compared with a lower SF, when sending the same amount of data, a higher SF requires a longer transmission time, which is called time-on-air. Theoretically, each step up in SF doubles the time required to transmit the same amount of data. More time-on-air means that the modem takes a longer time to launch an RF signal, and consumes more energy. A higher SF is not without advantages. A higher SF has more time-on-air, which means a higher chance of being received, which will increase the receiving sensitivity and thus achieve a longer transmission distance. Because of the orthogonal relationship between different SFs, the SF of the sender and receiver of the link must be known in advance. In addition, the Signal to Noise Ratio (SNR) at the input of the receiver must also be known. Signals can be received normally under negative SNR conditions, which improves the sensitivity, link budget, and coverage of the LoRa receiver.

The Bandwidth (BW) refers to the range of the upper-line frequency and lower-limit frequency through which a signal can pass. It can be understood as a channel, and only frequency signals within the signal bandwidth are allowed to pass. $\mathrm{BW}\in \left[7.8\;\mathrm{kHz},500\;\mathrm{kHz}\right]$, and the commonly used values are 125, 250, and 500 kHz [24]. In LoRa, the chirp rate only depends on the bandwidth, so increasing the BW can increase the effective data rate, but the sensitivity will also be affected, because the time-on-air is reduced and the probability of being correctly sampled is reduced. The bandwidth (BW) of LoRa is related to the symbol rate (Rs) and the data rate (DR). Rs can be calculated by Equation (1), which means that one chip is sent per Hz per second. We can obtain DR from Equation (2).

(1) $$R_{s}=\mathrm{BW}/\left(2^{\mathrm{SF}}\right)$$

(2) $$\mathrm{DR}=\mathrm{SF}*\left(\frac{\mathrm{BW}}{2^{\mathrm{SF}}}\right)*\mathrm{CR}$$

The reason why channel coding can detect and correct errors in the received bitstream is that some redundant bits are added to spread the information carried in a few bits to more bits. However, the node needs to transmit more bits than it should. To further improve the robustness of the link, the LoRa modem uses cyclic error correction coding for forwarding error detection and error correction. After using such error correction coding, transmission overhead will occur. In the presence of interference, forward error correction (FEC) can effectively improve the reliability of the link. The Coding Rate (CR) is the FEC rate used by LoRa modems, which provides protection against burst interference, and it can be set to 4/5, 4/6, 4/7, or 4/8 [29,30,31]. 

The results of the different combinations of BW and SF are shown in Table 1 using the SX1272 LoRa Calculator [32] published by Semtech. SF means the number of chips sent per symbol. As the SF value increases, the time-on-air also increases. At the same time, the probability that LoRa signals can be detected and received increases, and the transmission distance increases. BW determines the size of the chirp rate. As the BW value increases, the data rate will increase. However, as the time-on-air is reduced, the sensitivity will be affected. The size of the CR is related to the redundant information in the data packet. Therefore, a smaller value of CR is more resistant to interference, i.e., a packet transmitted with a code rate of 4/8 will be more tolerant to interference than a signal transmitted with a code rate of 4/5. Therefore, after theoretical analysis, we already know that SF, BW, and CR will affect the performance of LoRa. 

## 4. Study on the Relationship between Physical Layer Parameters and the Performance of LoRa

**Figure 2:**
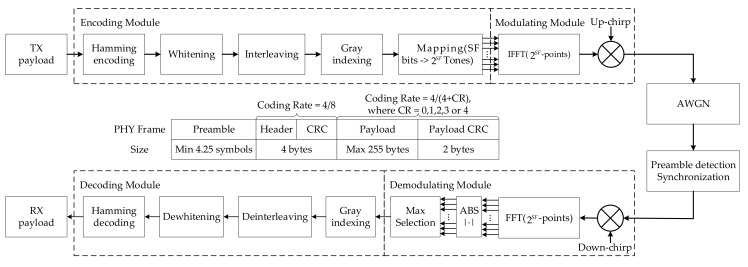
System model of the LoRa physical layer.

**Figure 3:**
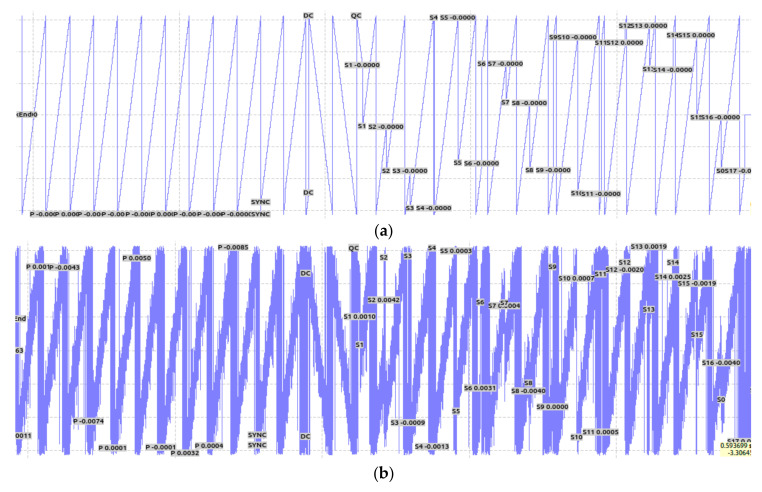
A time versus frequency diagram of the LoRa signal. (**a**) No noise in the channel. (**b**) The SNR in the channel is −3 dB.

A numerical model of the physical layer of an LoRa system is shown in Figure 2. This model is mainly used to simulate and evaluate the performance of an LoRa communication system through the AWGN channel. The LoRa PHY frame structure is the preamble part, which is used to synchronize the receiver with the incoming data stream. In the default explicit mode of operation, the number of bytes in the header part specifies the forward error correction (FEC) code rate, payload length, and 2 byte cyclic redundancy check (CRC) in the frame. This part allows the receiver to discard packets with invalid headers. The header part is coded at a code rate of 4/8, and the code rate of the payload is specified in the PHY header. The payload part is the data to be transmitted and the CRC part is used to check byte errors for cyclic redundancy and protect the payload. The input bits are first encoded using a Hamming code. Then, the bit stream is whitened, interleaved, and Gray indexed, and all SF bits are mapped to $2^{\mathrm{SF}}$ bits. Figure 1 presents a time versus frequency diagram of the LoRa waveform. The extended bits are further transferred to the Invert Fast Fourier Transformation (IFFT). Finally, the time domain signal is modulated by the chirp signal. The signal arrives at the receiving end after passing through the evaluation channel, and synchronization, frequency offset estimation, and compensation are performed before demodulation. Figure 3a illustrates the time versus frequency diagram when there is no noise in the channel. The time versus frequency diagram when the SNR is −3 dB in the AWGN channel is shown in Figure 3b. After the received signal is de-chirped, and then sent to the Fast Fourier Transform (FFT), the maximum value is found from all the values, and the maximum value is the demodulation result. Finally, Gray indexing, de-interleaving, de-whitening, and Hamming decoding are carried out to recover the information.

### 4.1. Brief Description of the Transceivers Used in the Experiments

**Figure 4:**
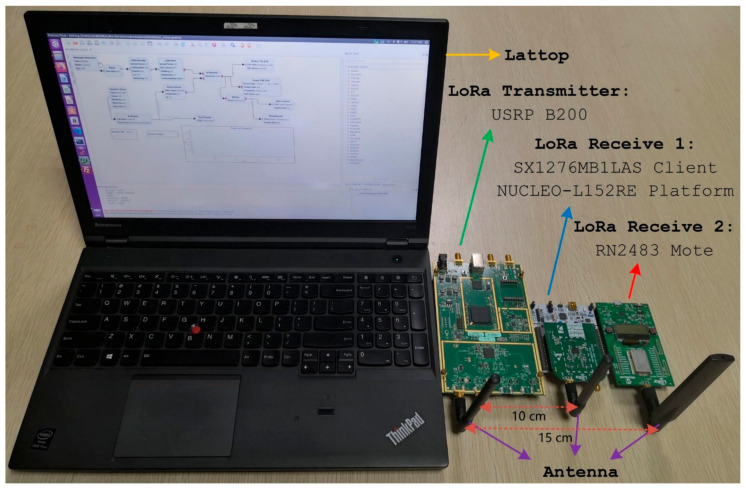
Our own developed LoRa transceiver and two commercial LoRa transceivers. From left to right: Lattop, a transceiver developed based on USRP B200 software radios; the Semtech SX1276 LoRa ™ mbed ™ Enabled Shields; and the Microchip Technology RN2483 LoRa^®^ Mote.

In order to evaluate the impact of parameter settings on the performance of LoRa, we used three LoRa devices in the experiments: An LoRa transceiver developed based on software radios, and two commercial transceivers, as shown in Figure 4. We designed the LoRa transceiver according to the characteristics and transmission process of the LoRa signal. This transceiver was developed based on USRP B200 software radios and can realize LoRa signals with various transmission characteristics. Our own developed LoRa transceiver uses a single antenna with 2 dBi gain. Moreover, our LoRa transceiver supports six different spreading factors, $\mathrm{SF}\in \left[7,12\right]$. The available BW are 125, 250, 500 kHz, etc., and CR are 4/5, 4/6, 4/7, and 4/8. By setting these parameters, we can obtain the LoRa signal we want. Our LoRa transceiver has no special requirements for terminal nodes and can work with any existing LoRa nodes.

The other two commercial transceivers are the Semtech SX1276 LoRa ™ mbed ™ Enabled Shields and the RN2483 Mote from Microchip. Semtech SX1276 LoRa ™ mbed ™ Enabled Shields combine the Semtech SX1276 Low Power Long Range Transceiver with the ARM mbed ™ Internet of Things (IoT) Device Platform. The mbed shields plug into mbed microcontroller development platforms from STMicroelectronics, NXP, and others. The SX1276MB1MAS is designed around ISM frequency bands 433 and 868 MHz for use in China and Europe. SX1276MB1MAS can operate in several frequency bands using two different RF ports: PA_LF and PA_HF. PA_LF covers the lower frequency band (band below 525 MHz), while PA_HF covers the higher frequency band (band above 860 MHz). The operating frequency band is controlled by register settings to produce the following values: 434, 470, 490, 868, and 915 MHz. However, changing the frequency band also requires the corresponding impedance matching in the RF front end to be changed to prevent reflections from degrading the performance. The transmit power of SX1276MB1MAS can reach 14 dBm, and the maximum power can reach 20 dBm by modifying the register and current limiter. Microchip Technology RN2483 LoRa^®^ Mote is an LoRaWAN Class A terminal device based on the RN2483 LoRa module. RN2483 Mote includes a light and temperature sensor that can generate data, which can start transmission according to a fixed schedule or by pressing. The RN2483 Mote operates from 863,000 to 870,000 MHz and 433,050 MHz to 434,790 MHz. The transmit power is 10 dBm on the 433 MHz band (limited to meet regulations) and the maximum transmit power is 14 dBm on the 868 MHz band. RN2483 Mote supports six different spreading factors, $\mathrm{SF}\in \left[7,12\right]$. The operating radio bandwidths are 125, 250, and 500 kHz.

### 4.2. LoRa Evaluation Experiment Setup

**Figure 5:**
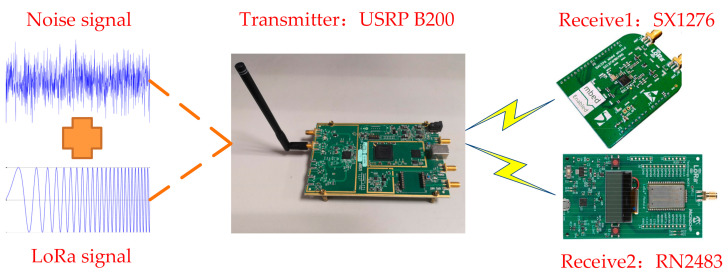
USRP B200 software radios were used to generate signals, which were superimposed by the LoRa signal and noise signal. SX1276 and RN2483 were used to receive signals.

The carrier frequency specified by transmitters and the receiver operates at 868 MHz. Transmitters can send LoRa packets, including a preamble with 10 up-chirps, an Start Frame Delimiter (SFD) with 2.25 down-chirps, and the payload with a random number of up-chirps. We used the UHD + GnuRadio library and developed our own LoRa Transceiver. We used white Gaussian noise as interference. The USRP B200 software radio generates noise and the LoRa signal and sends them 10,000 times continuously, as shown in Figure 5. At the same time, we used SX1276 and RN2483 to receive the signal. Additionally, the interval between data packets must not be less than three times the duration of the packet transmission. This constraint is imposed to ensure that each data packet has a long time window, without being disturbed, and the time is long enough to allow the message to be completely transmitted. The power value of the LoRa signal remains unchanged. We changed the amplitude of the noise signal, thereby changing the Signal to Noise Ratio (SNR). The SNR range was from −24 to 0 dB. We also saved the presence of cyclic redundancy check (CRC) errors in the received packets in our traces. Experiments changed the noise amplitude and the SF, BW, and CR of the LoRa signal to evaluate the packet reception performance of the LoRa according to the obtained Packet Error Ratio (PER) and SNR. We also measured the impact of different packet lengths on the packet reception performance of LoRa. The noise intensity of the experiment was increased by 1 dB until the Packet Error Ratio was 1. Therefore, all the measurement parameters in the experiment consisted of three settings for $\mathrm{BW}\in \left\{125\;\mathrm{kHz},250\;\mathrm{kHz},500\;\mathrm{kHz}\right\}$, six for $\mathrm{SF}\in \left[7,12\right]$, four for $\mathrm{CR}\in \left\{4/5,\;4/6,\;4/7,\;4/8\right\}$, and four for the $\mathrm{packet}\;\mathrm{length}\in \left\{4,\;32,\;128,\;255\right\}$ bytes.

## 5. Results and Analysis

### 5.1. The Impact of SF on the Packet Reception Performance of LoRa

In this experiment, we studied the LoRa packet reception performance with different SF values. We used the software radio platform to effectively generate LoRa signals with noise and accurately control each parameter. We let each data packet have a fixed sending cycle. All data packets were generated by random sequences. First, we kept the SF, BW, CR, and packet length unchanged, and gradually increased the noise intensity value, until the PER was equal to 1, and all the results were obtained. The information included SNR and PER. Then, the SF value was increased by one, the BW and packet length remained unchanged, and the noise intensity value was gradually increased until the PER was equal to 1. In this way, the experiment was conducted with all SF values. 

**Figure 6:**
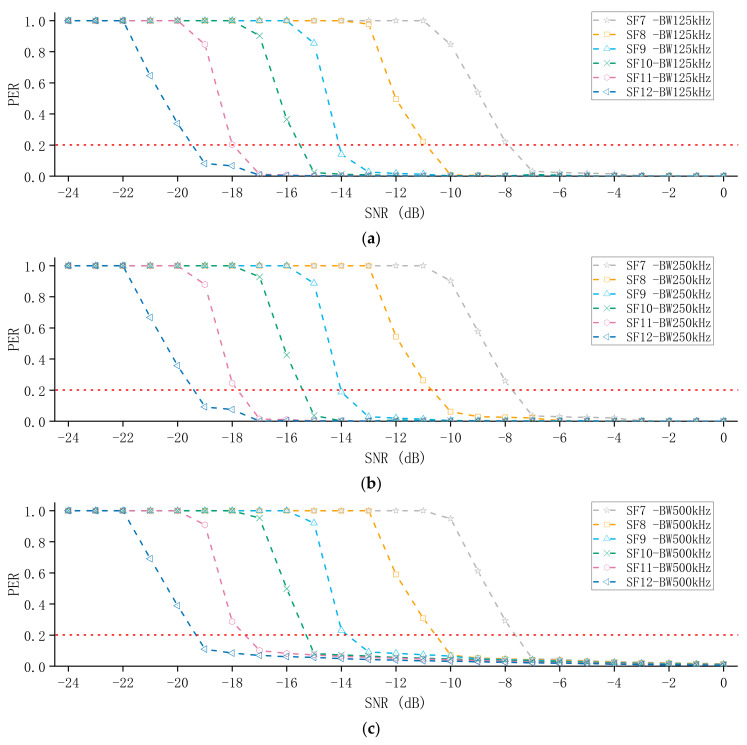
The measurement results of the impact of different SFs on the packet reception performance of LoRa: (**a**) BW = 125 kHz, measurement results of different SFs; (**b**) BW = 250 kHz, measurement results of different SFs; (**c**) BW = 500 kHz, measurement results of different SFs.

Figure 6 shows the SNR and PER for different SF values, as well as the distribution of the experimental results, rather than just the median and mean values. One can easily notice a certain regularity in PER behavior as a function of SNR, which allows us to make two conclusions:

(i) As the SNR decreases (the noise intensity becomes larger), the PER value increases, that is, the noise signal covers the LoRa signal. The LoRa signal is distorted and demodulation errors occur. In addition, as the SF value increases, the PER value becomes smaller. Therefore, a higher SF has better noise immunity. For example, for SNR = −12 dB, when SF = 7, PER = 1, but when SF = 12, PER = 0.034.
(ii) Each time SF increases by one, SNR in the high PER area (PER ≈ 1) decreases by 2 or 3 dB. For instance, from SF = 9 to SF = 10 for BW = 125 kHz, the high PER region’s SNR changes from −16 to −18 dB.

In addition, we also studied the influence of SF on the packet reception performance of LoRa when the BW is different. It can be seen from Figure 6 that when the BW is different, SF still has a great influence on the packet reception performance of LoRa.

### 5.2. The Impact of BW on the Packet Reception Performance of LoRa

**Figure 7:**
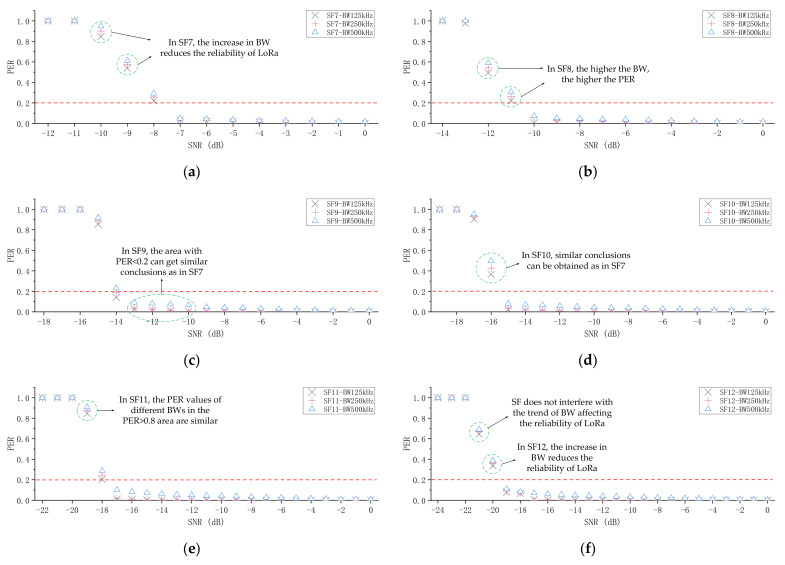
The measurement results of the influence of different BWs on the packet reception performance of LoRa when keeping the SF unchanged. (**a**–**f**) shows the PER for different BWs.

Next, we will discuss the impact of BW on the packet reception performance of LoRa. The method is roughly the same as that employed in the previous experiment. In this experiment, we kept the SF unchanged and observed the effect of BW on the packet reception performance of LoRa. From the previous analysis, we know that the chirp rate only depends on the bandwidth, so increasing the BW can increase the effective data rate, but the sensitivity will also be affected, because the time-on-air is reduced and the probability of being correctly sampled is reduced. Figure 7 shows the SNR and PER for different BWs. It is not difficult to draw the conclusion that as BW increases, PER also increases. 

Moreover, SNR can be changed not only by means of lowering the interferer power, but also by changing the BW. It appears that each downscaling of BW by a factor of two (e.g., from 500 to 250 kHz) causes the SNR to increase by 3 dB. At the same time, we also found that if BW is reduced by two times, the low PER area (PER < 0.2) will be extended one unit to the right along with SF. For example, when BW = 500 kHz, the low PER area is only at SF = 12; when BW = 250 kHz, the low PER area is at SF = 11 and 12; and when BW = 125 kHz, the low PER area is at SF = 10, 11, and 12. 

### 5.3. The Impact of CR on the Packet Reception Performance of LoRa

**Figure 8:**
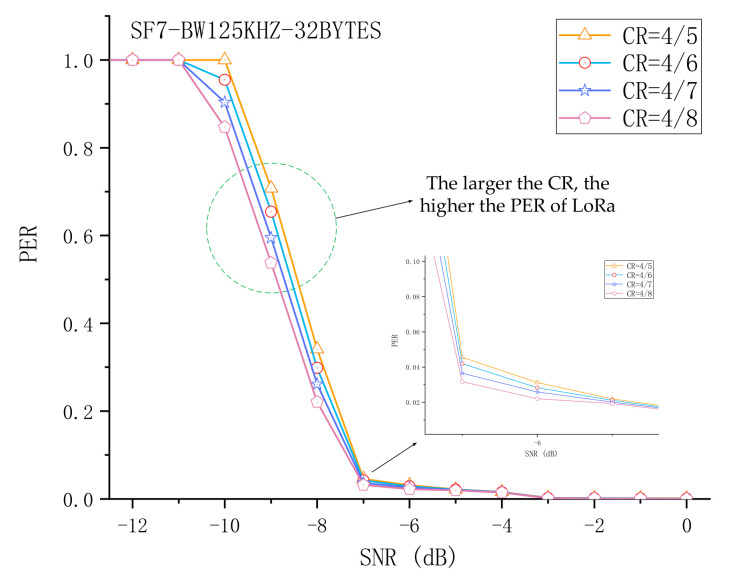
Experimental evaluation of the impact of CR on the packet reception performance of LoRa.

To improve the anti-interference ability, LoRa has added forward error correction (FEC) technology to the data transmission. The method used is to add redundant bits in the data bits, that is, 4 bits of data are added to the redundant bits to form 5, 6, 7, or 8 bits of data. In other words, if the coding rate is 4/n (n is equal to 5, 6, 7, or 8), the encoder generates a total of n bits of data, of which 4 bits are useful information and n − 4 is redundant. The Coding Rate (CR) can be set to 4/5, 4/6, 4/7, or 4/8. Although this method improves the reliability of communication, its disadvantage is also obvious, that is, the transmission time increases. We conducted extensive experiments on the different CRs, and the experimental results found that, as expected, as CR decreases, PER also decreases. In addition, it is worth noting that only under a little strong interference (PER > 0.5) will it be obvious that the difference in CR leads to a difference in PER. For example, the experimental settings included SF = 7, BW = 125 kHz, and a packet length of 32 bytes. When SNR = 0 dB, the PER of CR = 4/5 and 4/8 is 0.0013 and 0.0004, respectively. However, when SNR = −9 dB, the PER of CR = 4/5 and 4/8 is 0.7077 and 0.5380, respectively. Figure 8 shows the results of the impact of different CRs on the packet reception performance of LoRa.

### 5.4. The Impact of the Packet Length on the Packet Reception Performance of LoRa

Noise interference makes data packets more susceptible to individual bit errors. However, the link or transport layer protocol relies on checksums to detect errors in the packets and discard any packets that contain one or more error bits. Therefore, bit errors may cause a decrease in the communication quality, increase the packet loss rate, or increase the transmission delay. However, different packet lengths have different responses to noise interference. Long packets are more susceptible to interference, but short packets have to pay more for the header. Therefore, optimization of the packet length is an important research topic in wireless communication. In this section, we study the impact of the packet length on the packet reception performance of LoRa. A theoretical analysis on the impact of the length of the packet was conducted, and related experiments were then designed for evaluation.

#### 5.4.1. Theoretical Analysis of the Impact of the Packet Length

The previous experimental results tell us that within a given SNR, several parameters can be selected to achieve normal decoding of the signal, that is, the receiver has a normal decoding SNR range. Specifically, if the SNR of the received signal is less than the normal decoded SNR threshold, or the LoRa signal collides with the interference signal, the packet will be discarded due to a decoding error. This Packet Error Ratio can be expressed using Equation (3) [33]:

(3) $$p_{f}\left(L,SNR\right)=1-(1-p_{c}\left(L\right)\cdot \left(1-p_{s}\left(L,SNR\right)\right),$$

where $p_{c}\left(L\right)$ refers to the Packet Error Ratio caused by collision interference between packets; $p_{s}\left(L,SNR\right)$ refers to the probability of error caused by noise interference, that is, the SNR of signal is lower than the threshold; and L is the packet length.

For providing a simpler understanding of the situation when the SNR of the received signal is less than the SNR threshold for normal decoding, transmission errors caused by packet collisions are ignored in this paper, so Equation (3) can be transformed into Equation (4). The probability of successful reception $p_{sp}\left(L,SNR\right)$ is represented by Equation (5).

(4) $$p_{f}\left(L,SNR\right)=p_{s}\left(L,SNR\right)$$

(5) $$p_{sp}\left(L,SNR\right)=1-p_{s}\left(L,SNR\right)$$

For a given number of information bits contained in each data packet, $p_{sp}\left(L,SNR\right)$ can be used to calculate the expected data volume of each data packet (denoted as D below).

(6) $$D=L\cdot p_{sp}\left(L,SNR\right)=L-L\cdot p_{s}\left(L,SNR\right)$$

We assume that data packets are lost due to bit errors, and data bit errors are bursty and follow the Poisson process. The probability of at least one burst bit error in an Ɩ bits data packet is $P\left(Ɩ\right)$, which can be represented by Equation (7).

(7) $$P\left(Ɩ\right)\;=\;b+\left(1-b\right)\cdot \left(1-\exp\left(-\lambda Ɩ\right)\right)$$

Here, 1/λ is the average number of bits between two error bursts. The b is the possibility that the packet transmission may start during the error burst. 

If we define c = 1 − b, Equation (7) can be simplified as (8):

(8) $$P\left(Ɩ\right)\;=\;1-cƖ\exp\left(-\lambda Ɩ\right)$$

If the packet loss rates of two different packet lengths Ɩ1 and Ɩ2 are known, then we can use Equation (9) and (10) to find the parameters c and λ.

(9) $$\lambda =\ln\left(\frac{1-P\left({Ɩ}_{1}\right)}{1-P\left({Ɩ}_{2}\right)}\right)/\left({Ɩ}_{2}-{Ɩ}_{1}\right)$$

(10) $$c=\frac{1-P\left({Ɩ}_{1}\right)}{\exp\left(-\lambda {Ɩ}_{1}\right)}=\frac{1-P\left({Ɩ}_{2}\right)}{\exp\left(-\lambda {Ɩ}_{2}\right)}$$

Knowing λ and c, we can estimate the loss rate of different packet lengths to make adjustments to the transmission mechanism, and the retransmission mechanism should also be considered. We assume that it can be retransmitted indefinitely. In theory, long packets are more susceptible to interference than short packets, so more retransmissions may be required. However, if a node has a message (i.e., 128 bytes) to send, it can send a packet carrying the 128-byte message directly or divide this message into four 32-byte blocks. This means that short packets need to send more headers, which is the cost of short packets.

#### 5.4.2. The Result of the Impact of the Packet Length on the Packet Reception Performance of LoRa

**Figure 9:**
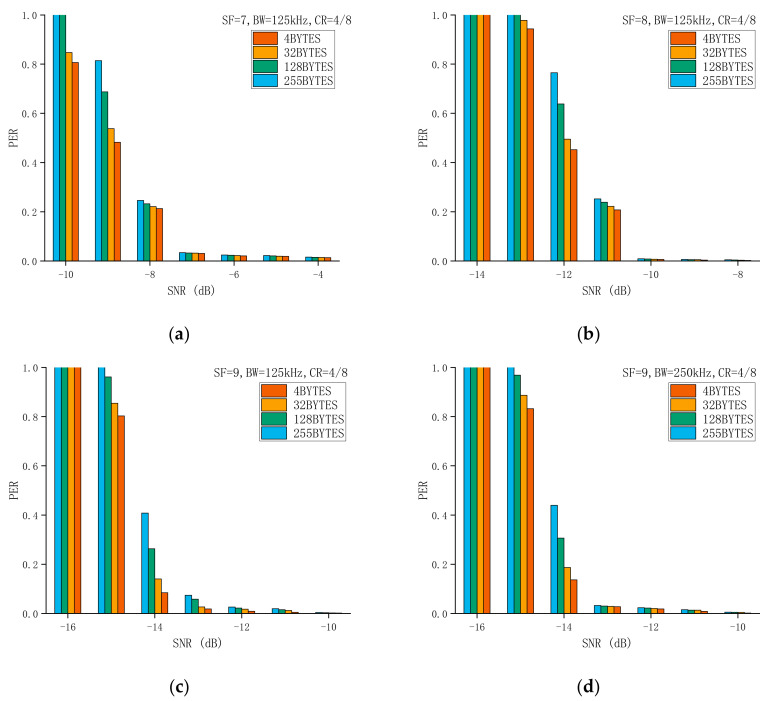

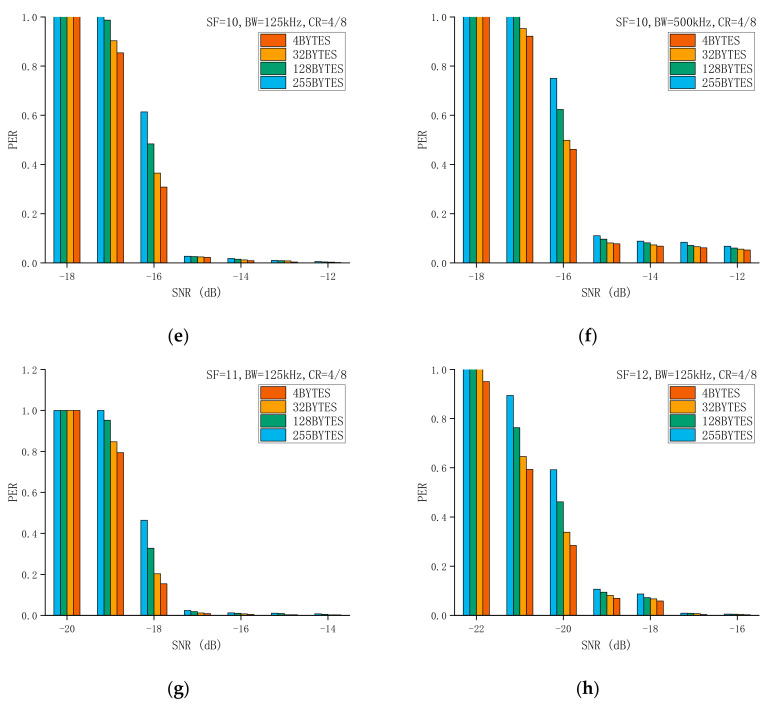
The impact of different packet lengths on the packet reception performance of LoRa. (**a**–**h**) shows the results of the Packet Error Ratio for different packet lengths.

We conducted extensive experiments based on the previous experimental settings, and the packet length was 4, 32, 128, and 255 bytes. There was enough time between the two data packets for the receiver to fully receive and decode the data. The conclusions we obtained on the impact of SF and BW on the packet reception performance of LoRa are still valid. Our current focus is mainly on the impact of the packet length on the packet reception of LoRa. Figure 9 shows the results of the Packet Error Ratio for different packet lengths. We provide detailed experimental results, including the performance evaluation of different packet lengths under different SF and BW conditions. We used the experimental results obtained to verify the theoretical derivation previously proposed and the results show that the theory is correct. Therefore, we can use the obtained value to derive the Packet Error Ratio of other packet lengths and optimize the transmission scheme. For example, when SF = 7, BW = 125 kHz, CR = 4/8, and SNR = −9 dB, the Packet Error Ratio of 4 bytes is approximately equal to 0.4826, and the Packet Error Ratio of 128 bytes is approximately equal to 0.6871. We can substitute these two results into Equations (9) and (10), and the results of λ and c are approximately equal to 0.0041 and 0.5288, respectively. Finally, the Packet Error Ratio of 128 bytes can be substituted into Equation (10) to figure out the Packet Error Ratio of 32 bytes. The result is approximately equal to 0.5362, and our actual measurement result is approximately equal to 0.5380. The experimental results are very close to the theoretical results, with a difference of only 0.0018. The experimental results fully demonstrate the validity of the theory. We used similar methods and different experimental results to conduct further verification, and finally drew the conclusion that the derivation of the equation is correct. Therefore, based on this theory, we can calculate the Packet Error Ratio of different packet lengths and measure whether the node needs to change the packet length. 

We also obtained other meaningful findings. On the one hand, longer data packets can better offset the overhead associated with the header, resulting in a higher number of correctly received and decoded data packets using long packets within the considered payload length range. On the other hand, increasing the length of data packets means a lower immunity to interference. Although long data packets have a longer time-on-air, the time is mainly consumed by the encoded data, so it does not make it easier to be received by the receiver. In addition, it is worth noting that in areas where the communication link is unreliable (PER>0.2), the performance difference of different packet lengths is obvious, while in other areas (PER≈0), the Packet Error Ratio of different packet lengths is similar. As shown in Figure 9b, when SF = 8 and SNR = −12 dB, the PER of 255 bytes is 0.7652, and the PER of 4 bytes is 0.4521. However, when SNR = −8 dB, the PER of 255 and 4 bytes is 0.0047 and 0.0021, respectively. In SF10 and BW500 kHz, similar conclusions to SF10 and BW125 kHz can be obtained, as shown in Figure 9e,f. Therefore, BW does not interfere with the trend of the impact of the packet length on the reliability of LoRa. As shown in Figure 9a–h, SF does not interfere with the trend of the impact of the packet length on the reliability of LoRa.

### 5.5. Measurement Results of Different LoRa Receivers

**Figure 10:**
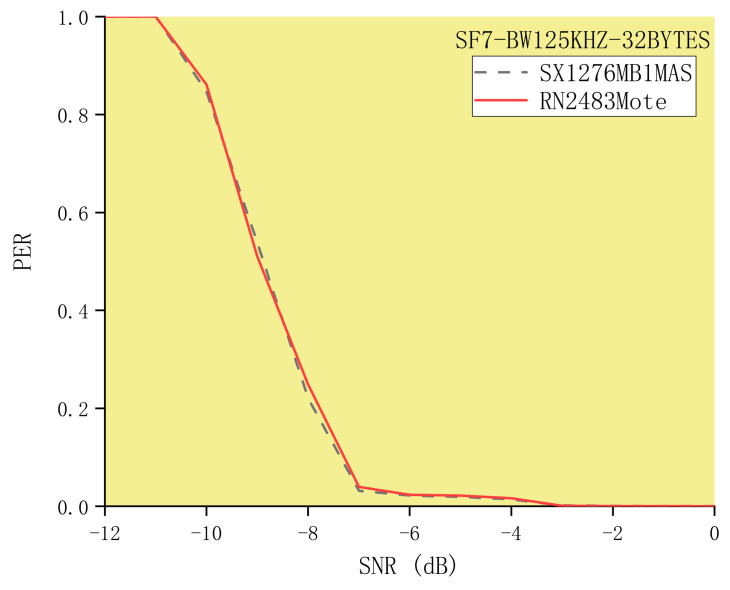
Measurement results of different LoRa receivers (SX1276MB1MAS and RN2483 Mote).

To reduce the influence of other factors, ensure the consistency and scientificity of the experimental results, and make the experimental results convincing, we used the USRP B200 software radios to continuously transmit LoRa signals 10,000 times and used two commercial devices (SX1276MB1MAS and RN2483 Mote) as receivers. It should be noted that all experiments in our paper used SX1276MB1MAS and RN2483 Mote as receivers. The experimental results show that the packet reception performance of SX1276MB1MAS is similar to that of RN2483 Mote. For example, when SF = 7, BW = 125 kHz, CR = 4/8, and the packet length is 32 bytes, the results of the Packet Error Ratio of SX1276MB1MAS and RN2483 Mote are similar, as shown in Figure 10. In other words, the experiments on evaluating the impact of the packet reception performance of LoRa have little to do with LoRa receivers produced by different manufacturers.

### 5.6. Parameter Setting of the Transmission Scheme 

Energy is critical for communication networks that require low power consumption. In fact, there has been a lot of work on energy-saving communication protocols and power management strategies, and related work is still ongoing. The time required for successful communication and energy consumption are positively correlated, so we will reduce the energy consumption of nodes from the perspective of reducing the time for successful communication. 

The previous experiment tells us the significance of each physical parameter for the performance of LoRa, and also gives a detailed Packet Error Ratio $p_{f}$ report. Assuming that the Packet Error Ratio remains unchanged and the number of possible retransmission attempts is unlimited, the average amount of retransmission required for each packet to be successfully received is shown in Equation (11).

(11) $$r=\overset{\infty }{\underset{n=1}{\sum }}p_{f}^{n}=\frac{p_{f}}{1-p_{f}}$$

**Figure 11:**
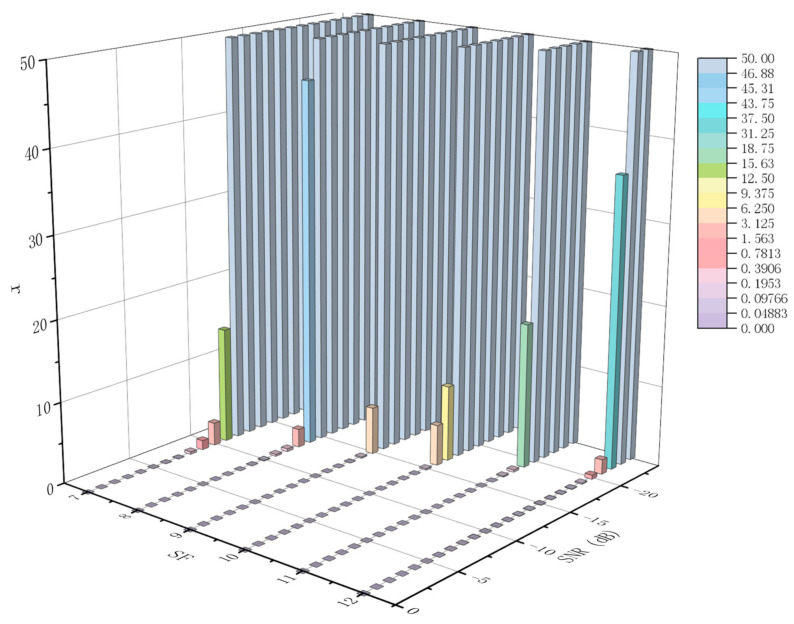
The number of packet retransmissions for different Signal to Noise Ratio (SNR) and SF settings.

Figure 11 shows the number of packets that need to be retransmitted under different SNRs and SFs. The *x* axis represents different SF values, $\mathrm{SF}\in \left[7,12\right]$; the *y* axis represents different SNR values, $\mathrm{SNR}\in \left[-24,0\right]$ dB; and the z axis represents the number of packets that need to be retransmitted. At the same time, we assume that BW is 125 kHz, CR is 4/8, and the packet length is 32 bytes. When the Packet Error Ratio is equal to 1, the LoRa signal is completely covered by the noise signal, and the receiver cannot detect the preamble signal and decoded data. At this time, the number of packets that need to be retransmitted should be infinite, and we use r = 50 to represent it.

In order for the receiver to have enough time to receive the LoRa signal completely and to be able to estimate the additional delay caused by the retransmission, we constrain the packet interval to three packet transmission times. Therefore, the time $t_{r}$ required to retransmit a data packet can be obtained. Moreover, if parameters such as SF, BW, CR, and the packet length are set, the transfer time $t_{0}$ and Packet Error Ratio $p_{f}$ are obtained. Since the time $t_{r}$ required for a packet to be retransmitted is four times the transfer time $t_{0}$, the time $t_{r}$ required for a packet to be retransmitted is also obtained. The time t required for the packet to be successfully received can be obtained from Equation (12).

(12) $$t=\;t_{0}+t_{r}\cdot \frac{p_{f}}{1-p_{f}}$$

**Figure 12:**
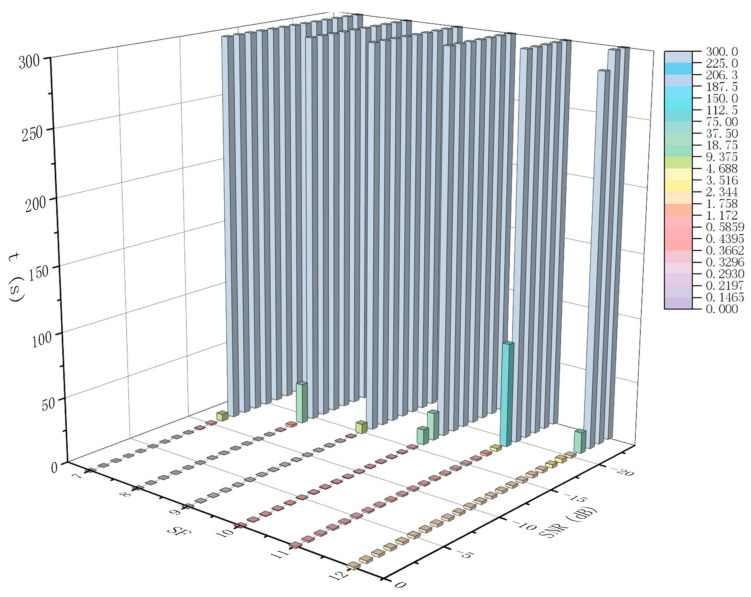
Transmission time t for different SNR and SF settings. The lower the (t), the better.

Assuming the previous conditions and that the number of retransmissions is unlimited, the time t required for the packet to be successfully received is shown in Figure 12. It can be seen from the figure that, although a higher SF has better immunity to interference, it requires a much more successful transmission time than a lower SF under certain SNR conditions. For example, when SNR = −13 dB, BW = 125 kHz, CR = 4/8, and the packet length is 32 bytes, the successful transmission time t = 0.3459 s with SF = 9 is much shorter than t = 1.9814 s with SF = 12.

Furthermore, although the transmission time $t_{0}$ and retransmission time $t_{r}$ under different parameter settings are different, when the parameters are set, the normal $t_{0}$ of the packet is a constant without retransmission, and the $t_{r}$ is also a constant, so the only $p_{f}$ is related to the total required successful transmission time t. We can reduce the required t by reducing $p_{f}$. We know that the size of $p_{f}$ is related to SF, BW, CR, and the packet length. We can reduce the $p_{f}$ by adjusting these parameters. However, it should be noted that the $t_{0}$ and $t_{r}$ are also related to the above parameters. By adjusting these parameters, $p_{f}$ is reduced, but $t_{0}$ and $t_{r}$ may increase, resulting in an increase in the t. Therefore, we need to find the optimal parameter configuration to balance these variables, so as to obtain the minimum time t required for the packet to be successfully received. We use examples to illustrate how to adjust these parameters to obtain the minimum t. In order to make our proposed scheme easy to understand, we first clarify how to optimize the SF to reduce the t. Then, multiple parameters are comprehensively considered to obtain the minimum transmission time t, thereby reducing the energy consumption of successful communication, and finally achieving a balance of reliability, delay, and energy consumption. We assume that the SNR is −9 dB, the BW is 125 kHz, the CR is 4/8, and the packet length is 32 bytes, and then adjust the SF to reduce t. Meanwhile, in order to ensure that the receiver has a sufficient receiving time window without being interfered with by other data packets, we restrict the interval time of data packets so that it is not less than 3 times of the packet transmission time. We substitute the previous experimental results under this condition into Equation (12), and obtain the t at different SFs, as shown in Table 2. The result shows that t is the smallest when SF is 8. Although SF = 8 has a longer transmission time $t_{0}$ than SF = 7, the Packet Error Ratio of SF = 8 is much smaller than SF = 7. In other words, SF = 8 will also have fewer retransmissions, thus shortening the time t required for the packet to be successfully received. Moreover, although the Packet Error Ratios for SF = 9, 10, 11, and 12 are small, the number of retransmissions required will be small. However, their Packet Error Ratio is not that different from SF = 8, and their transmission time $t_{0}$ is much greater than SF = 8, so it takes more time for the packet to be successfully received. Imagine that if we still use SF7 without adjusting the SF, the time t for successful communication will be three times as long as the SF adjusted. If the most sensitive parameter configuration (SF12) is adopted directly, the time required is 11 times that after adjustment.

**Figure 13:**
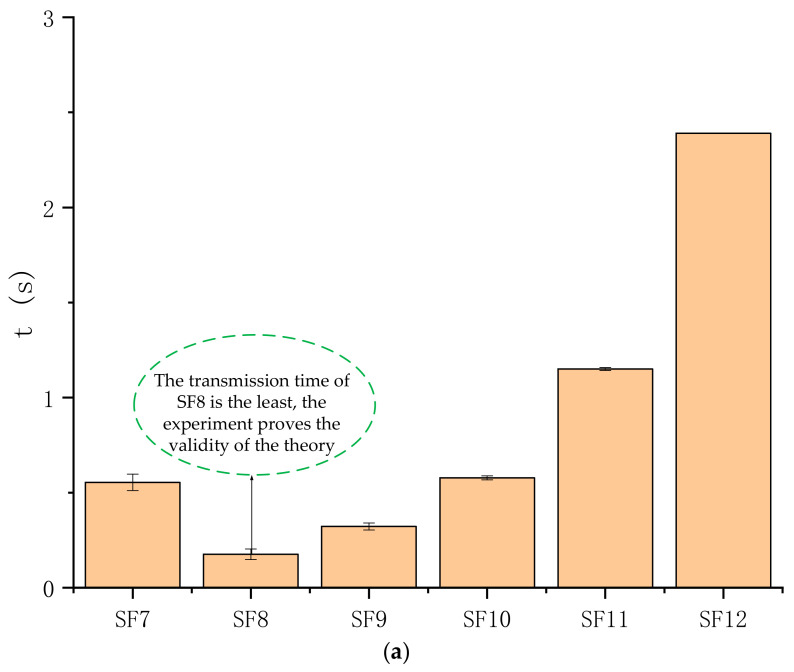

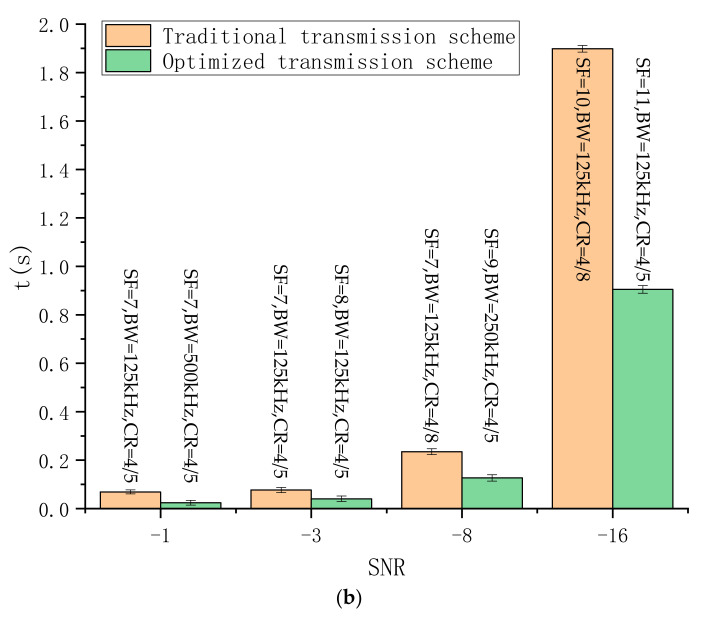
Transmission time t for different parameter configurations. The lower the (t), the better. (**a**) shows result of the average time required for each packet to be successfully transmitted; (**b**) shows the result of the transmission time when the packet length is 32 bytes with different SNRs.

In order to test the effectiveness of this method, we first set the required initial conditions, then used the USRP transmitter to continuously send 1000 packets, and repeated the experiment five times. The interval between packets is the time of three packets. Therefore, the result of the average time required for each packet to be successfully transmitted is shown in Figure 13a. As expected, the time required for SF8 is the smallest value, which is the same as the result we obtained through theoretical calculations. The comprehensive consideration of multiple parameters is the same as the previous method. The PER of all parameters corresponding to the SNR is substituted into Equation (12) to obtain the shortest successful transmission time t. For example, when the SNR is −3 dB, we optimized the configuration of SF, CR, BW, and the packet length to reduce the communication time, thereby reducing the energy consumption of the node. Since short packets will send more headers than long packets, there is no need to divide long packets into short packets. We assumed that the packet length of the data to be sent is 32 bytes, and substituted the PER under different parameters into Equation (12). The results show that the successful transmission time is the shortest when the node is configured with SF = 7, BW = 250 kHz, and CR = 4/5. We also proved the correctness of the conclusion through experiments. Figure 13b shows the result of the transmission time when the packet length is 32 bytes with different SNRs. Compared with the original parameter configuration, the communication time after parameter optimization is reduced by about 50%.

## 6. Future Work

At present, the practical application of LoRa has achieved remarkable results in many fields, such as manufacturing, agriculture, home furnishing, transportation, car networking, and medical treatment, resulting in an increasing density and scale of networked sensor deployments. However, LoRa networks usually use a star topology, with thousands of LoRa nodes connected to a single LoRa gateway. For example, in a smart factory, multiple sensor nodes send the collected information to an LoRa gateway. Such an aggregated network structure will cause serious data packet collisions, resulting in a large amount of data packet loss and a drop in throughput. The failure of communication also increases the delay, and energy is wasted. Furthermore, if network scheduling is used for information transmission, it will bring about the problem of hidden terminals, which will also cause collisions. Since LoRa is the long-distance transmission, this problem is even more obvious. Meantime, the network scheduling scheme will undoubtedly increase the complexity of the network and consume more energy of the nodes. Last but not least, some applications require concurrent transmission, that is, multiple points instantaneously transmit messages to the gateway. For example, when a poisonous gas leak triggers an alarm in a factory, all poisonous gas detection nodes in the factory are required to immediately upload current air status information. Therefore, research on the concurrent transmission is also necessary. In the future, we expect to evaluate the LoRa performance in concurrent transmission scenarios. In addition, we hope to find a solution to achieve multi-packet decoding, which can ultimately increase the throughput of the LoRa network and reduce latency. 

## 7. Conclusions

It is believed that a better understanding of the relationship between physical layer parameters and the packet reception performance of LoRa can help deal with technical challenges, such as reliability, delay, and energy consumption. Therefore, we conducted extensive experimental evaluations on the influence of physical layer parameters on the packet reception performance of LoRa. The experimental results found the following: (i) If the SF value is increased by one, the SNR of the high PER region (PER ≈ 1) will decrease by 2 or 3 dB; (ii) every time the BW value doubles, the range of the reliable communication area (PER ≈ 0% interference immunity area) will decrease by one SF index and the SNR will also be reduced by 3 dB; and (iii) compared with the impact of SF, although CR has a smaller impact, it is still important in reducing PER. We also propose a PER expression for different packet lengths to analyze the impact of the packet length on the packet reception performance of LoRa. The extensive experimental results confirm the validity of the theory and illustrate that the packet length only has a significant effect on the packet reception performance of LoRa at the edge of the communication link. Meanwhile, the experimental results also suggest that if a node needs to send a large amount of data, the node should use a long packet transmission scheme. Compared with long packets, short packets mean more headers are sent, which will consume more transmission time and energy. Finally, we have discussed how to combine parameters based on the experimental results, and reduce energy consumption by shortening the time for successful communication, thereby balancing reliability, delay, and energy consumption. The solution we propose can quickly combine parameters according to the SNR to meet the requirements of different scenarios and shorten the communication time by about 50% compared with the traditional transmission solution, which greatly reduces the energy consumption.

## Figures and Tables

**Table 1:** Main transmission features for the different Spreading Factors (SFs) and Bandwidths (BWs).

SF	BW	Time-on-Air (msec)	Data Rate (kbps)	Sensitivity (dBm)
7	125	44.29	5.47	−123
7	250	22.14	10.94	−120
7	500	11.07	21.88	−117
8	125	88.58	3.13	−126
8	250	44.29	6.25	−123
8	500	22.14	12.50	−120
9	125	144.38	1.76	−129
9	250	72.19	3.52	−126
9	500	36.01	7.03	−123
10	125	288.77	0.98	−132
10	250	144.38	1.95	−129
10	500	72.19	3.91	−126
11	125	577.54	0.54	−134.5
11	250	288.77	1.07	−131.5
11	500	144.38	2.15	−128.5
12	125	1155.07	0.29	−137
12	250	577.54	0.59	−134
12	500	288.77	1.17	−131

**Table 2:** Given some parameters, the result of the transmission time t with different SF values.

**SF**	**PER**	$t_{0}\;(\mathrm{ms})$	$t_{r}\;(\mathrm{ms})$	t
7	0.5380	96.51	386.04	546.05
8	0.0050	176.64	706.56	180.19
9	0.0019	320.51	1282.04	322.95
10	0.0011	575.49	2301.96	578.02
11	0.0003	1150.98	4603.92	1152.36
12	0.0000	2039.81	8159.24	2039.81

## Data Availability

The data presented in this study are available on request from the corresponding author. The data are not publicly available due to [the project is still in progress and the data needs to be kept confidential].

## Abbreviations

The following abbreviations are used in this manuscript:

IoT	Internet of Things
SF	Spreading Factor
BW	Bandwidth
CR	Coding Rate
SFD	Start Frame Delimiter
PER	Packet Error Ratio
SNR	Signal to Noise Ratio
CSS	Chirp Spread Spectrum
LPWAN	Low Power Wide Area Network
IFFT	Inverse Fast Fourier Transform
FFT	Fast Fourier Transform
Rs	Symbol Rate
DR	Data Rate
CRC	Cyclic Redundancy Check
FEC	Forward Error Correction
